# Nasal Rifampicin Improves Cognition in a Mouse Model of Dementia with Lewy Bodies by Reducing α-Synuclein Oligomers

**DOI:** 10.3390/ijms22168453

**Published:** 2021-08-06

**Authors:** Tomohiro Umeda, Yukari Hatanaka, Ayumi Sakai, Takami Tomiyama

**Affiliations:** Department of Translational Neuroscience, Osaka City University Graduate School of Medicine, 1-4-3 Asahimachi, Abeno-ku, Osaka 545-8585, Japan; umemaru@med.osaka-cu.ac.jp (T.U.); m20mc009@yf.osaka-cu.ac.jp (Y.H.); sakai@icb.med.osaka-u.ac.jp (A.S.)

**Keywords:** rifampicin, intranasal administration, α-synuclein, oligomer, dementia with Lewy bodies, prevention

## Abstract

α-Synuclein oligomers are thought to play an important role in the pathogenesis of dementia with Lewy bodies (DLB). There is no effective cure for DLB at present. Previously, we demonstrated that in APP- and tau-transgenic mice, oral or intranasal rifampicin reduced brain Aβ and tau oligomers and improved mouse cognition. In the present study, we expanded our research to DLB. Rifampicin was intranasally administered to 6-month-old A53T-mutant α-synuclein-transgenic mice at 0.1 mg/day for 1 month. The mice displayed memory impairment but no motor deficit at this age, indicating a suitable model of DLB. α-Synuclein pathologies were examined by the immunohistochemical/biochemical analyses of brain tissues. Cognitive function was evaluated by the Morris water maze test. Intranasal rifampicin significantly reduced the levels of [pSer129] α-synuclein in the hippocampus and α-synuclein oligomers in the visual cortex and hippocampus. The level of the presynaptic marker synaptophysin in the hippocampus was recovered to the level in non-transgenic littermates. In the Morris water maze, a significant improvement in spatial reference memory was observed in rifampicin-treated mice. Taken together with our previous findings, these results suggest that intranasal rifampicin is a promising remedy for the prevention of neurodegenerative dementia, including Alzheimer’s disease, frontotemporal dementia, and DLB.

## 1. Introduction

Neurodegenerative diseases with the accumulation of α-synuclein aggregates are collectively called α-synucleinopathy and include Parkinson’s disease (PD), dementia with Lewy bodies (DLB), and multiple system atrophy (MSA) [1]. In PD and DLB, α-synuclein accumulates in neurons to form Lewy bodies and Lewy neurites in a specific brain region. The main clinical symptom of PD is the motor dysfunction Parkinsonism, which is characterized by resting tremors, akinesia, bradykinesia, muscular rigidity, and postural instability, due to dopaminergic neuronal loss in the nigrostriatal system. The main symptom of DLB is cognitive impairment, such as hallucinations, due to neuronal alterations in the visual cortex of the occipital lobe. As the disease progresses, the α-synuclein pathology spreads throughout the brain, and both PD and DLB eventually show Parkinsonism and dementia. In MSA, which conceptually integrates several disorders, including olivo-ponto-cerebellar atrophy (MSA-C), striato-nigral degeneration (MSA-P), and Shy–Drager syndrome, α-synuclein accumulates in oligodendrocytes to form glial cytoplasmic inclusions. The eventual clinical symptoms of MSA are cerebellar ataxia, Parkinsonism, and dysautonomia due to neurodegeneration in the cerebellum, brainstem, and spinal cord.

There is no effective cure for α-synucleinopathy at present. Following the trend of Aβ and tau immunotherapy, vaccines, and antibodies for α-synuclein have been developed and undergone clinical trials [2] (for the latest news about these clinical trials, see the Alzforum website: https://www.alzforum.org/therapeutics/search?fda_statuses=&target_types%5B%5D=33416&therapy_types%5B%5D=161&therapy_types%5B%5D=162&conditions=&keywords-entry=&keywords=#results (accessed on 5 August 2021)). Some of these vaccines and antibodies are designed to recognize oligomeric forms of α-synuclein, which are considered more pathogenic than fibrillar forms or inclusions [3,4]. We previously demonstrated that a well-known antibiotic, rifampicin, inhibits the oligomerization of not only Aβ and tau but also α-synuclein in vitro [5]. When orally administered to APP- and tau-transgenic (Tg) mice, rifampicin reduced Aβ and tau oligomers in the brain and improved cognition of the mice [5]. These results suggest that rifampicin is also effective at rescuing motor and/or cognitive dysfunction in model mice of α-synucleinopathy by reducing brain α-synuclein oligomers. In fact, one study showed that when intraperitoneally administered to a mouse model of MSA that expresses α-synuclein in oligodendrocytes, rifampicin reduced the amount of monomeric and oligomeric α-synuclein and suppressed neurodegeneration in the brain [6].

However, oral rifampicin occasionally induces adverse effects such as liver dysfunction and drug–drug interactions (https://www.drugs.com/mtm/rifampin.html (accessed on 5 August 2021)). These undesired actions are considered to occur during the drug’s first pass from the intestine to the liver. We assumed that these adverse effects could be avoided by administering the drug intranasally, because the drug injected into the nasal cavity is efficiently delivered to the brain by passing through the nasal mucosa epithelium and transported less to the liver [7]. Thus, we previously tested the intranasal administration of rifampicin in APP-Tg mice and found that it caused less hepatopathy than oral rifampicin and had higher efficacy in cognitive improvement [8]. Although the data obtained in animals may not be directly applicable to humans, intranasally administered oxytocin has been shown to enter the brain in functionally relevant amounts in humans [9]. These findings indicate that intranasal administration is the best route for rifampicin as a brain-targeting drug in the treatment of neurodegenerative diseases. Thus, in the present study, we examined the therapeutic effects of nasal rifampicin on α-synuclein oligomers and associated neuronal dysfunction in a mouse model of DLB.

## 2. Results

**Figure 1:**
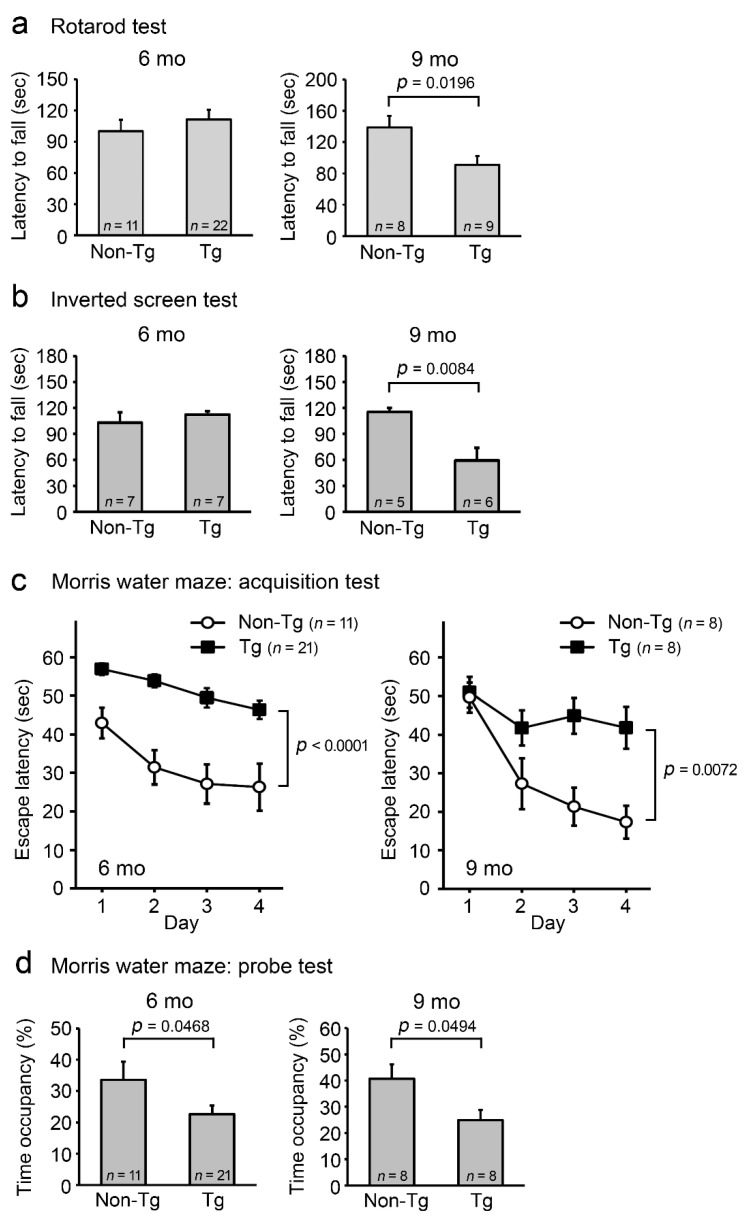
Motor and cognitive functions in A53T-mutant α-synuclein-Tg mice. Male and female littermates were subjected to the rotarod (**a**), inverted screen (**b**), and Morris water maze test (**c**,**d**) at 6 and 9 months old (mo). The numbers of mice used are shown in each figure. (**a**) In the rotarod test, Tg mice first displayed a motor disturbance at 9 months. (**b**) This trend was confirmed by the inverted screen test using different individuals of age-matched littermates. In the Morris water maze test, memory acquisition (**c**) and retention (**d**) of Tg mice were significantly impaired as early as 6 months.

As a model of DLB, we used Huα-Syn(A53T) transgenic mouse line G2-3. This line was originally generated as a model of PD and has been reported to develop adult-onset neurodegeneration with a progressive motor dysfunction leading to death [10]. To evaluate its suitability for a DLB model, we initially studied age-dependent pathological phenotypes of the mice. Motor and cognitive functions of the heterozygous littermates were evaluated in our laboratory by the rotarod, inverted screen, and Morris water maze tests at ages 6 and 9 months. In the rotarod test, Tg mice displayed no abnormalities at 6 months, but some disturbances at 9 months (Figure 1a). This trend was confirmed by the inverted screen test using different individuals of age-matched littermates (Figure 1b). After the rotarod test, the mice were subjected to the Morris water maze test. Memory acquisition (Figure 1c) and retention tests (Figure 1d) revealed that the spatial reference memory of Tg mice was impaired as early as 6 months and also at 9 months. These results indicate that in this mouse line, cognitive dysfunction precedes motor deficit. Our findings are consistent with a previous report showing that Tg mice displayed cognitive deficits at 6 months in the Barnes maze test [11] and developed motor signs at 9–16 months (https://www.jax.org/strain/006823 (accessed on 5 August 2021)).

**Figure 2:**
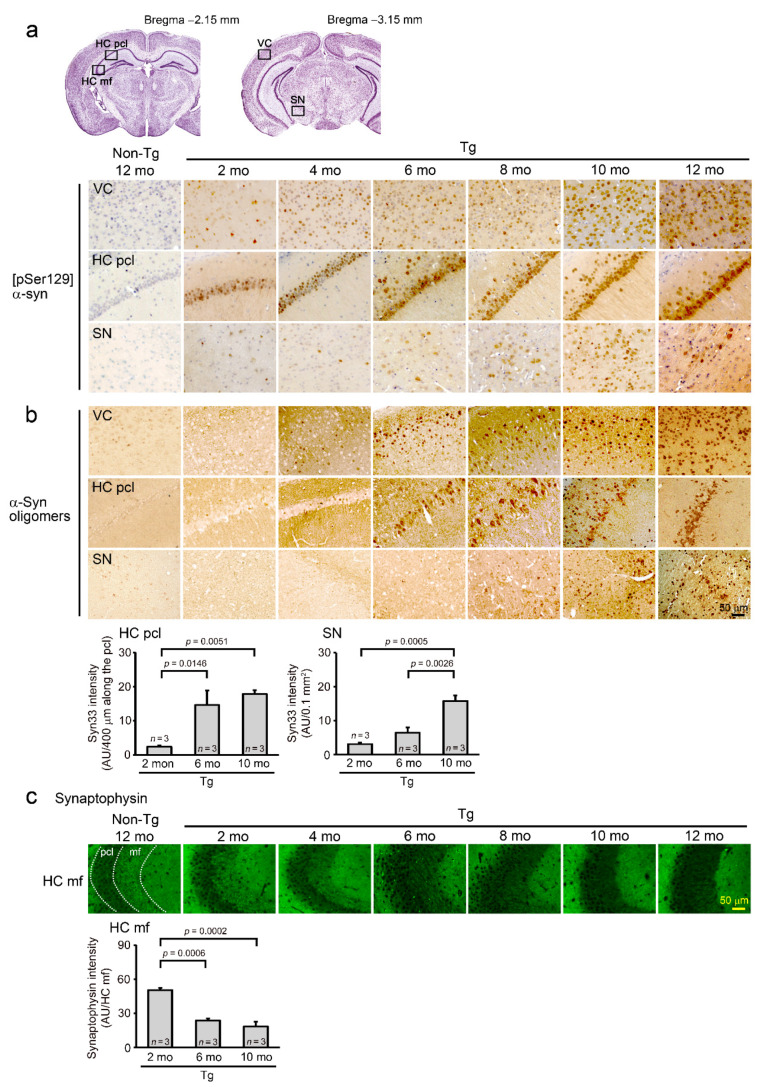
α-Synuclein pathologies in Tg mice. Brain sections prepared at 2, 4, 6, 8, 10, and 12 months old (mo) were stained with antibodies to [pSer129] α-synuclein (**a**), α-synuclein oligomers (**b**), and synaptophysin (**c**). (**a**) [pSer129] α-synuclein was detected in the visual cortex (VC) and hippocampal pyramidal cell layer (HC pcl) as early as 2 months and in the substantia nigra (SN) at 6 months. (**b**) α-Synuclein oligomers first appeared in the VC at 4 months. The accumulation became evident in the HC pcl at 6 months and in the SN at 10 months. Quantification of α-synuclein oligomers was performed in the HC and SN at 2, 6, and 10 months. Syn33 is an antibody to α-synuclein oligomers. (**c**) Synaptophysin in the hippocampal mossy fibers (HC mf) began to decrease at 4 months and were markedly attenuated at 6 months. Dotted lines indicate the boundaries of the HC pcl and HC mf regions. Quantification of synaptophysin was performed in the HC mf at 2, 6, and 10 months using the intensity in the HC pcl as a control.

**Figure 3:**
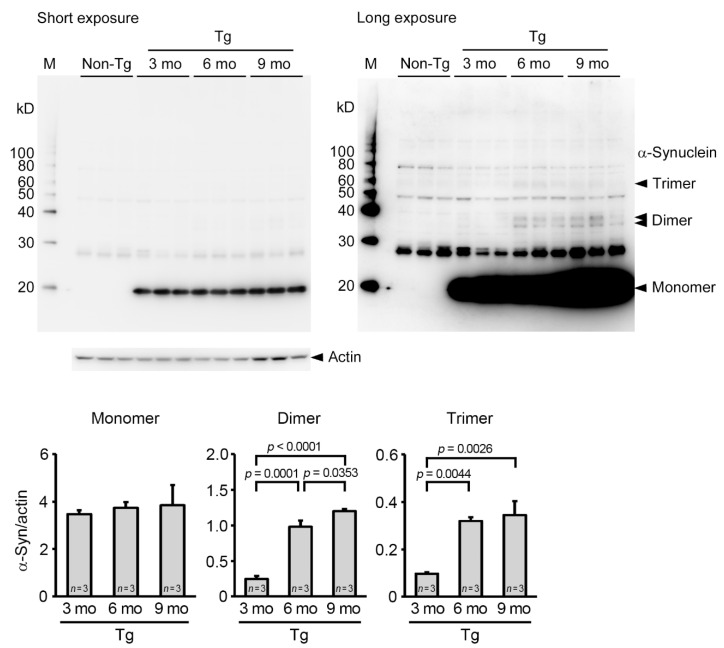
Accumulation of α-synuclein oligomers in Tg mice. Brain soluble fractions were prepared at 3, 6, and 9 months old (mo) and examined for α-synuclein oligomers by Western blotting. Non-Tg littermate samples were obtained at 6 months. α-Synuclein dimers and trimers were significantly increased at 6 months. M, MagicMark XP Western Protein Standard (Invitrogen, Carlsbad, CA, USA).

Next, we examined α-synuclein pathologies by immunohistochemistry. Brain sections were prepared from mice 2-, 4-, 6-, 8-, 10-, and 12-months old and stained with antibodies to phosphorylated and oligomeric α-synuclein and presynaptic marker synaptophysin. [pSer129] α-synuclein was detected in the visual cortex and hippocampus as early as 2 months and in the substantia nigra at 6 months (Figure 2a). α-Synuclein oligomers first appeared in the visual cortex at 4 months and were clearly detected in the visual cortex and hippocampus at 6 months. The accumulation of α-synuclein oligomers in the substantia nigra was faintly observed at 8 months but became evident at 10 months (Figure 2b). Synaptophysin in the hippocampal CA3 region began to decrease at 4 months and markedly attenuated at 6 months (Figure 2c). These pathological changes increased in an age-dependent manner. We also examined α-synuclein oligomers in brain soluble fractions at 3, 6, and 9 months of age by Western blotting. Dimers and trimers of α-synuclein significantly increased at 6 months in Tg mice (Figure 3). These observations led us to speculate that in Tg mice, cognitive impairment is caused by a cortical and hippocampal accumulation of α-synuclein oligomers, whereas motor dysfunction is elicited by their nigral accumulation. At the same time, we confirmed that this mouse line at 6–8 months can be used as a model of DLB that shows only cognitive abnormalities, and after 9 months, they can be used as a model of PD with dementia or DLB with Parkinsonism that exhibits both cognitive and motor dysfunction.

**Figure 4:**
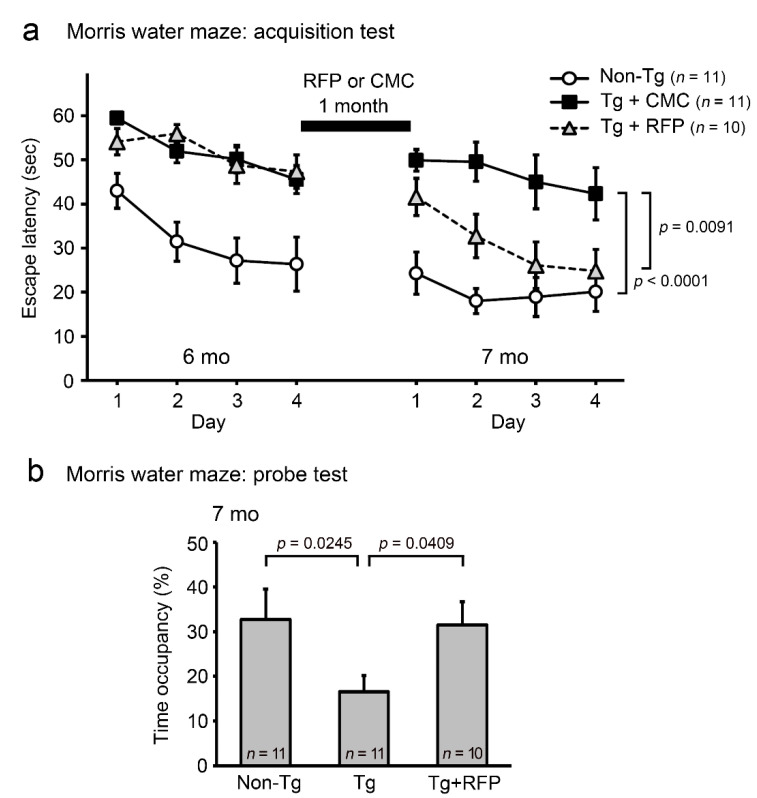
Effect of intranasal rifampicin on the cognition of Tg mice. After the Morris water maze test in Figure 1c–d, 6-month-old (mo) Tg mice were divided into 2 groups. One group was intranasally treated with rifampicin (RFP) at 0.1 mg/day for 1 month and the other was treated with CMC. Non-Tg littermates were treated with CMC only. After treatment, a second water maze test was performed. The numbers of mice assigned are shown in each figure. (**a**) Rifampicin significantly improved the memory of Tg mice. The curves on the left prior to rifampicin treatment (6 months) are reproduced from the data shown in Figure 1c. (**b**) Memory retention was also ameliorated by rifampicin treatment.

Thus, we decided to use younger mice to investigate the therapeutic potential of rifampicin against DLB. After the first Morris water maze task in Figure 1c–d, 6-month-old mice were divided into two groups. One group received intranasal rifampicin at 0.1 mg/day for 1 month and the other received carboxymethylcellulose (CMC). Then, cognition and α-synuclein pathologies were evaluated by the second water maze test and subsequent immunohistochemical staining. As shown in Figure 4a, non-Tg littermates maintained their spatial reference memory even after 1 month. Tg mice, which failed to establish memory of the platform location in the first water maze test, retained their impaired memory function upon CMC treatment. In contrast, the Tg mice that received nasal rifampicin showed a significant improvement to almost the same level as non-Tg littermates at day 4. The memory retention of Tg mice was also ameliorated by rifampicin treatment (Figure 4b).

**Figure 5:**
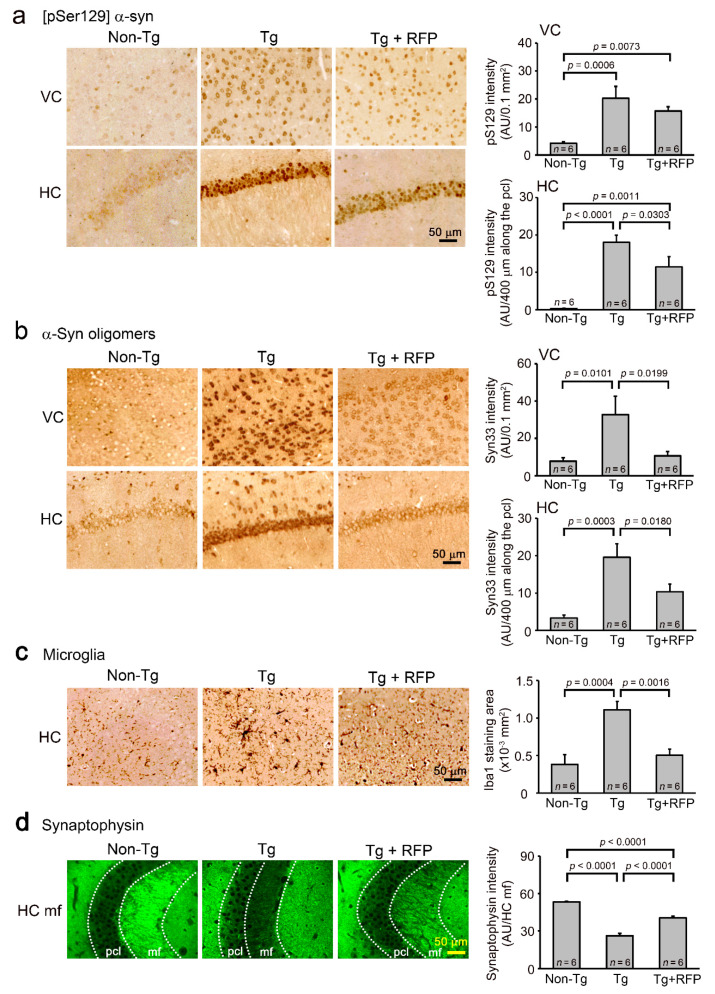
Effects of intranasal rifampicin on α-synuclein pathologies in Tg mice. After the second Morris water maze test, brain sections were prepared and stained. The levels of [pSer129] α-synuclein (**a**) and α-synuclein oligomers (**b**) in the visual cortex (VC) and hippocampus (HC) were quantified. (**a**,**b**) [pSer129] α-synuclein was significantly reduced in the HC and to some extent in the VC by rifampicin treatment, while α-synuclein oligomers were significantly decreased in both regions. Syn33 is an antibody to α-synuclein oligomers. (**c**) The levels of activated microglia were quantified in the HC. Rifampicin significantly attenuated microglial activation. (**d**) Synaptophysin levels in hippocampal mossy fibers (HC mf) were measured using the intensity in the pyramidal cell layer (pcl) as a control. Rifampicin significantly recovered the synapse level in Tg mice. RFP, rifampicin; AU, arbitrary units.

**Figure 6:**
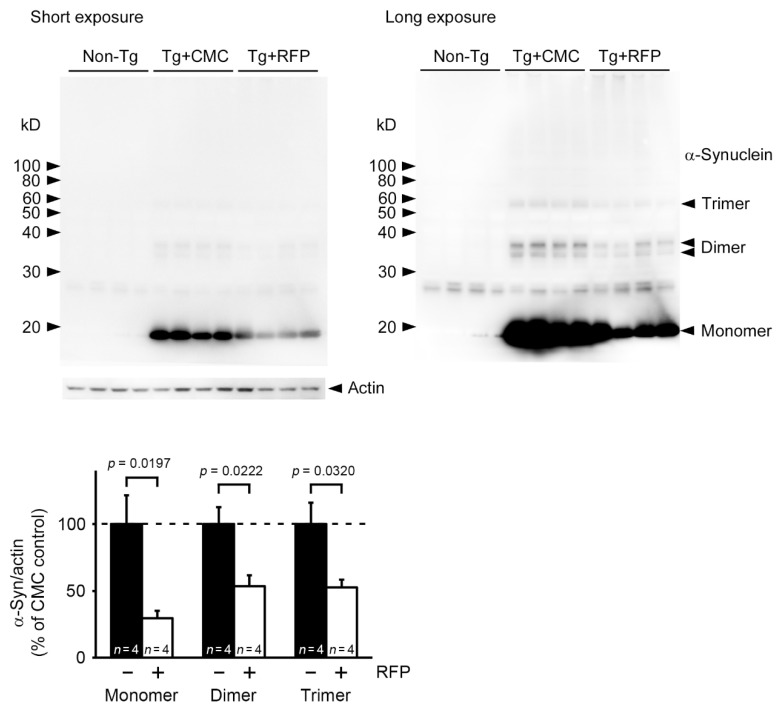
Clearance of α-synuclein oligomers by intranasal rifampicin (RFP). After the second Morris water maze test, brain soluble fractions were prepared and subjected to Western blot analysis. The density of visualized α-synuclein bands were quantified and compared between CMC- and rifampicin-treated Tg mice. α-Synuclein monomers, dimers, and trimers were reduced by rifampicin treatment in Tg mice. + and − mean rifampicin- and CMC-treatment, respectively.

Immunohistochemistry showed that the level of [pSer129] α-synuclein was significantly reduced by rifampicin treatment in the hippocampus and tended to decrease in the visual cortex (Figure 5a). The level of α-synuclein oligomers significantly decreased in both regions (Figure 5b). We also examined microglial activation, an index of brain inflammation. Rifampicin significantly attenuated the levels of activated microglia in the hippocampus (Figure 5c). Rifampicin also recovered the synaptophysin level in the hippocampal mossy fibers to that of non-Tg littermates (Figure 5d). Western blot analysis revealed that rifampicin treatment reduced the levels of α-synuclein monomers, dimers, and trimers in brain soluble fractions (Figure 6). Together, these results indicate that nasal rifampicin is effective at improving cognition by reducing brain α-synuclein oligomers in a mouse model of DLB.

## 3. Discussion

The anti-amyloidogenic property of rifampicin was first described by us in 1994 [12], in which we showed that rifampicin was effective at inhibiting Aβ aggregation in vitro. This action was likely associated with its scavenging ability against hydroxyl free radicals [13]. Based on these findings, a clinical trial with oral rifampicin and doxycycline was carried out in Canada for mild to moderate Alzheimer’s disease. Unfortunately, no beneficial effects on cognition or function were observed [14]. However, this result does not necessarily indicate the ineffectiveness of rifampicin. Clinical studies on Aβ immunotherapy have revealed that Aβ-targeting medicines successfully cleared Aβ plaques from patients’ brains but failed to improve their cognition [15,16]. The reasons for this failure are attributed to the timing of medication, which was after the neurodegeneration had significantly worsened [16,17] and to the wrong drug target. Accumulating evidence has indicated that the real culprit in Alzheimer’s disease is soluble Aβ oligomers rather than insoluble fibrils, such as plaques [18,19]. Previously, we found that rifampicin inhibited the oligomerization of Aβ, tau, and α-synuclein in vitro and that when administered orally, rifampicin improved the cognition of APP- and tau-Tg mice by reducing Aβ and tau oligomers [5]. These results imply that when started before the neurodegeneration, rifampicin treatment could prevent cognitive decline. A recent retrospective study in non-demented patients with Hansen’s disease indicated that rifampicin is effective at preventing Alzheimer’s disease, but this requires at least 450 mg daily for 1 year [20].

Since our 1994 paper, the inhibition of α-synuclein aggregation by rifampicin has been reported by other researchers [21]. Furthermore, the therapeutic effects of rifampicin were examined in a mouse model of MSA, where intraperitoneally administered rifampicin reduced brain monomeric and oligomeric α-synuclein and suppressed neurodegeneration [6]. Subsequently, a clinical trial with oral rifampicin was performed in patients with MSA [22]. However, again, no beneficial effects were observed. We hypothesize the same reason as for Alzheimer’s disease: the timing of medication was too late [23].

Like Aβ, tau [24,25] and α-synuclein [3,4] have been suggested to become toxic after oligomerization. Several vaccines and antibodies targeting α-synuclein oligomers are undergoing clinical trials for PD and MSA (see the Alzforum website referred to in the Introduction). A recent study has shown that in Sigma-1 receptor knockout mice, intraperitoneal rifampicin reduced α-synuclein oligomers in the substantia nigra, prevented dopaminergic neuron loss, and improved motor coordination [26]. In the present study, we demonstrated that nasal rifampicin successfully reduced brain α-synuclein oligomers and improved cognition in a mouse model of DLB. Although the effect of rifampicin on motor dysfunction in PD mice remains to be studied, the aforementioned results in model mice implicate that rifampicin has therapeutic potential in the prevention of α-synucleinopathy. For long-term prophylactic treatment, intranasal administration would be better than oral or intraperitoneal administration in terms of safety, drug efficacy, and invasiveness [8]. Nevertheless, we should be cautious that chronic rifampicin administration may cause adverse events. Further investigation would be necessary to improve the safety of the drug.

It is noteworthy that rifampicin only inhibits the oligomerization of pathogenic, amyloidogenic proteins, including Aβ, tau, and α-synuclein, but not of physiologically assembling proteins such as glutathione-S-transferase [5]. In addition, rifampicin has not only anti-amyloidogenic but also antioxidant [13,27] and anti-inflammatory properties [28,29,30]. Oxidative stress and inflammation are profoundly involved in the pathogenesis of neurodegenerative diseases [31,32]. We showed that APP-, tau-, and α-synuclein-Tg mice all displayed microglial activation, which reflects brain inflammation, and rifampicin attenuated these pathological changes [5, and the present study]. Furthermore, oxidative stress and inflammation, along with glutamate-induced excitotoxicity, contribute to neuronal damage in ischemic stroke [33]. These phenotypes suggest that rifampicin is effective in both neurodegenerative and cerebrovascular diseases. In practice, intraperitoneal rifampicin has been shown to protect neurons from cerebral ischemia in mice [34]. The main obstacle for rifampicin as a brain-targeting medicine is its low penetration into the brain [35]. However, this problem can be solved by administering the drug intranasally, as shown in our previous study [8]. These features collectively indicate the usefulness of rifampicin as a neuroprotective agent in the treatment of brain disorders.

## 4. Materials and Methods

### 4.1. Mice

B6.Cg-2310039L15RikTg(Prnp-SNCA*A53T)23Mkle/J mice, also known as Huα-Syn(A53T) transgenic line G2-3, were purchased from the Jackson Laboratory (Bar Harbor, ME, USA). This mouse line was originally generated as a model of PD that expresses A53T-mutant human α-synuclein under the mouse prion protein promoter [9]. The Tg mice were mated with wild-type C57BL/6N mice in our animal facility and maintained as heterozygotes for the transgene.

### 4.2. Behavioral Tests

Male and female mice aged 6 and 9 months were examined for their motor and cognitive function. Motor function was initially assessed by the rotarod test using an MK-610A rotarod treadmill for mice (Muromachi Kikai, Tokyo, Japan). The mice were trained to stay on the rod rotating at 5 rpm for 180 s. Then, the rotation speed was increased from 4 to 40 rpm over 240 s, and this accelerating rotarod training was repeated 2 times. On the next day, the accelerating rotarod test was performed 2 times with a 1 h interval in between. The times when the mice fell off the rod were recorded. Cases when mice completely rotated down by clinging onto the rod were considered drops from the rod. The mean time of the 2 trials was calculated. Motor strength and coordination were further measured by the inverted screen test. The mice were placed individually on top of a circular wire screen (24 cm in diameter, openings 1.2 cm × 1.2 cm) mounted horizontally on a metal rod. The rod was then rotated 180° and the mice’s behavior was observed for 2 min. The times when the mice fell off the screen were recorded. When the mice climbed to the top of the screen, or remained clinging to the underside of the screen, 120 s of latency was assigned. The mean time of the 2 trials was calculated. Cognitive function was evaluated by the Morris water maze test, as described previously [36]. Mice were trained to swim to a hidden platform 5 times a day with 5 min intervals between each swim over 4 consecutive days. The times when the mice climbed on the platform were recorded. The mean time of the 5 trials was calculated each day. At day 5, the retention of spatial reference memory was assessed by a probe trial consisting of a 60 s free swim in the pool without the platform.

### 4.3. Rifampicin Treatment

After 6-month-old Tg mice performed the Morris water maze test, they were divided into two groups. One group was treated with rifampicin and the other with CMC every day from Monday to Friday, every week for 1 month. Rifampicin (Sigma-Aldrich, St. Louis, MO, USA) was dissolved to 10 mg/mL in 0.5% low-viscosity CMC (Sigma-Aldrich). A measure of 10 μL of rifampicin (i.e., 0.1 mg) or CMC solution was administered into the bilateral nasal cavity using micro tips, as described previously [8]. Non-Tg littermates were treated with CMC alone. Following the 1-month treatment, a second water maze test was performed. Rifampicin treatment was continued during the behavioral test. After the second water maze test, each Tg mouse group was divided into two groups: one group for immunohistochemical analysis and the other for biochemical analysis.

### 4.4. Immunohistochemical Analysis

To study age-dependent neuropathology in the model mice, brain sections of Tg and non-Tg littermates were prepared at ages 2, 4, 6, 8, 10, and 12 months, as described previously [36]. To expose the antigens, sections were boiled in 0.01 N HCl, pH2 for 10 min for phosphorylated α-synuclein or 10 mM citrate buffer, pH6 for 30 min for α-synuclein oligomers and synaptophysin. After blocking with 10% calf serum overnight, sections were stained with antibodies to [pSer129] α-synuclein (Abcam-Epitomics, Cambridge, UK), α-synuclein oligomers (Syn33; Merck Millipore, Darmstadt, Germany), and synaptophysin (SVP-38; Sigma-Aldrich), essentially as described previously [36]. The staining was followed by a biotin-labeled second antibody (Vector Laboratories, Burlingame, CA, USA), horseradish peroxidase (HRP)-conjugated avidin-biotin complex (Vector Laboratories), and an HRP substrate, diaminobenzidine (DAB; Dojindo, Kumamoto, Japan) for α-synuclein species or an FITC-labelled second antibody (Jackson Laboratory) to synaptophysin. The stained specimens were viewed under a BZ-X800 fluorescence microscope (Keyence, Osaka, Japan), and the images of certain brain regions were photographed. α-Synuclein pathologies and synapse loss were evaluated by quantifying the staining intensity in a constant area in each photograph using NIH ImageJ software (ImageJ bundled with 64-bit Java 1.8.0_172; https://imagej.nih.gov/ij/ (accessed on 5 August 2021)).

To examine the effects of rifampicin, brain sections of rifampicin- and CMC-treated mice were prepared after the second water maze test. The levels of [pSer129] α-synuclein, α-synuclein oligomers, and synaptophysin were estimated. Activated microglia were also stained with an antibody to Iba-1 (Fujifilm Wako, Osaka, Japan) after pretreatment of brain sections at pH6 for 30 min. Microglial activation was assessed by quantifying the staining area in each photograph.

### 4.5. Western Blot Analysis of α-Synuclein Oligomers

Brains were collected from Tg and non-Tg littermates at 3, 6, and 9 months after birth. Each cerebral hemisphere was homogenized by sonication in 5 volumes of Tris-buffered saline containing a protease inhibitor cocktail (P8340; Sigma-Aldrich) and a phosphatase inhibitor cocktail (Nacalai Tesque, Kyoto, Japan). The homogenates were centrifuged at 100,000× *g* for 30 min to separate insoluble materials. The supernatants were subjected to Western blot analysis with antibodies to α-synuclein (Syn211; Sigma-Aldrich) and actin (Sigma-Aldrich) followed by HRP-labelled second antibodies (Bio-Rad Laboratories, Hercules, CA, USA) and chemiluminescent HRP substrate (ImmunoStar LD; Fujifilm Wako). The stained α-synuclein and actin were visualized and quantified using an ImageQuant LAS 500 image analyzer (GE Healthcare, Hino, Japan).

To evaluate the effects of rifampicin, the brains of rifampicin- and CMC-treated mice were collected after the second water maze test. The levels of α-synuclein oligomers in the brain soluble fractions were determined by Western blotting.

### 4.6. Statistical Analysis

All experiments and data analyses were performed under unblinded conditions. A comparison of means among more than two groups was performed by ANOVA or two-factor repeated measures of ANOVA (for the Morris water maze test) followed by Fisher’s PLSD test. Differences with a *p*-value of <0.05 were considered significant.

## 5. Conclusions

Intranasal rifampicin improved the memory of 6-month-old A53T-mutant α-synuclein-Tg mice by reducing brain α-synuclein oligomers. Taken together with our previous findings in APP- and tau-Tg mice, the present results suggest that nasal rifampicin could be a promising remedy for the prevention of neurodegenerative dementia including Alzheimer’s disease, frontotemporal dementia, and DLB.

## Data Availability

The data presented in this study are available upon request.
